# Friedreich's ataxia induced pluripotent stem cell-derived cardiomyocytes display electrophysiological abnormalities and calcium handling deficiency

**DOI:** 10.18632/aging.101247

**Published:** 2017-05-30

**Authors:** Duncan E. Crombie, Claire L. Curl, Antonia JA Raaijmakers, Priyadharshini Sivakumaran, Tejal Kulkarni, Raymond CB Wong, Itsunari Minami, Marguerite V. Evans-Galea, Shiang Y. Lim, Lea Delbridge, Louise A. Corben, Mirella Dottori, Norio Nakatsuji, Ian A. Trounce, Alex W. Hewitt, Martin B. Delatycki, Martin F. Pera, Alice Pébay

**Affiliations:** 1 Centre for Eye Research Australia, Royal Victorian Eye and Ear Hospital, Melbourne, Australia; 2 Ophthalmology, Department of Surgery, the University of Melbourne, Melbourne, Australia; 3 Department of Physiology, the University of Melbourne, Melbourne, Australia; 4 O'Brien Institute Department, St Vincent Institute of Medical Research, Fitzroy, Australia; 5 Centre for Neural Engineering & Department of Electrical and Electronic Engineering, The University of Melbourne, Melbourne, Australia; 6 Institute for Integrated Cell-Material Sciences, Kyoto University, Kyoto, Japan; 7 Bruce Lefroy Centre for Genetic Health Research, Murdoch Childrens Research Institute, and Department of Paediatrics, The University of Melbourne, Melbourne, Australia; 8 School of Psychological Sciences, Monash University, Frankston, Australia; 9 Menzies Institute for Medical Research, School of Medicine, University of Tasmania, Hobart, Australia; 10 Victorian Clinical Genetics Services, Parkville, Australia; 11 Department of Anatomy and Neurosciences, the University of Melbourne, Florey Neuroscience & Mental Health Institute, Walter and Eliza Hall Institute of Medical Research, Australia

**Keywords:** Friedreich's ataxia, induced pluripotent stem cells, cardiomyopathy, modelling

## Abstract

We sought to identify the impacts of Friedreich's ataxia (FRDA) on cardiomyocytes. FRDA is an autosomal recessive degenerative condition with neuronal and non-neuronal manifestations, the latter including progressive cardiomyopathy of the left ventricle, the leading cause of death in FRDA. Little is known about the cellular pathogenesis of FRDA in cardiomyocytes. Induced pluripotent stem cells (iPSCs) were derived from three FRDA individuals with characterized GAA repeats. The cells were differentiated into cardiomyocytes to assess phenotypes. FRDA iPSC- cardiomyocytes retained low levels of FRATAXIN (FXN) mRNA and protein. Electrophysiology revealed an increased variation of FRDA- cardiomyocyte beating rates which was prevented by addition of nifedipine, suggestive of a calcium handling deficiency. Finally, calcium imaging was performed and we identified small amplitude, diastolic and systolic calcium transients confirming a deficiency in calcium handling. We defined a robust FRDA cardiac-specific electrophysiological profile in patient-derived iPSCs which could be used for high throughput compound screening. This cell-specific signature will contribute to the identification and screening of novel treatments for this life-threatening disease.

## INTRODUCTION

FRDA is an autosomal recessive degenerative condition with neuronal and non-neuronal manifestations [1]. Cardiomyopathy is detected in two thirds of individuals with FRDA [2–4]. Individuals with FRDA generally present with progressive cardiomyopathy of the left ventricle, which is the leading cause of death in FRDA due to arrhythmias and/or heart failure [5, 6]. Little is known of the cellular impacts of FRDA in the heart, but cardiomyocyte necrosis and cellular fibrosis have been identified [6, 7]. Systolic function generally remains normal until late in disease progression [8]. In approximately 96% of affected individuals, FRDA is due to homozygosity for an unstable expanded GAA repeat mutation in the first intron of *FXN* resulting in reduced expression of the nuclear-encoded mitochondrial protein FXN [9–11]. Despite the identification of FXN, its precise role in FRDA pathogenesis remains elusive and remarkably little is known about the molecular pathology of cardiomyocytes in FRDA [12].

Human iPSCs [13–15] have been derived from individuals with FRDA [12, 16–19]. Morphological abnormalities and a disorganized mitochondrial network in iPSC-derived- cardiomyocytes have been identified [16, 19]. There are no abnormalities under basal conditions, when cultivated in the presence of iron, cellular hypertrophy occurs [19]. However, more detailed functional studies are needed to characterize the cardiomyocytes from FRDA iPSCs. Here, we derived FRDA iPSCs to assess the electrophysiological and calcium (Ca^2+^) cycling properties of cardiomyocytes, to identify potential mechanisms underlying the cardiomyopathy observed in FRDA.

## RESULTS

### Generation of three FRDA-iPSC lines and differentiation into cardiomyocytes

We generated FRDA-iPSC lines from three individuals with different GAA length repeat numbers (Table 1). We also collected data regarding disease severity as measured by the Friedreich Ataxia Rating Scale (FARS) [20]. The FARS is scored out of 167, a higher score indicating greater disease severity. Clinical parameters of individuals from whom the lines were derived are as follows: FA6 (female; GAA1 1077, GAA2 1077; FARS score 96.5); FA8 (male; GAA1 476, GAA2 545; FARS score 64.5); FA9 (male; GAA1 733, GAA2 943; FARS score 118). The individuals with FRDA from which iPSCs were derived presented with the following cardiac phenotypes: FA6: normal ejection fraction (55%), normal ventricular wall thickness, mildly dilated left atrium (mild cardiomyopathy); FA8: normal ejection fraction (60%), moderate increase in relative wall thickness (RWT), severe dilatation of the left atrium (typical FRDA cardiomyopathy) and FA9: low normal ejection fraction (50%), increased RWT, borderline increase in left atrial size (typical FRDA cardiomyopathy).

**Figure 1:**
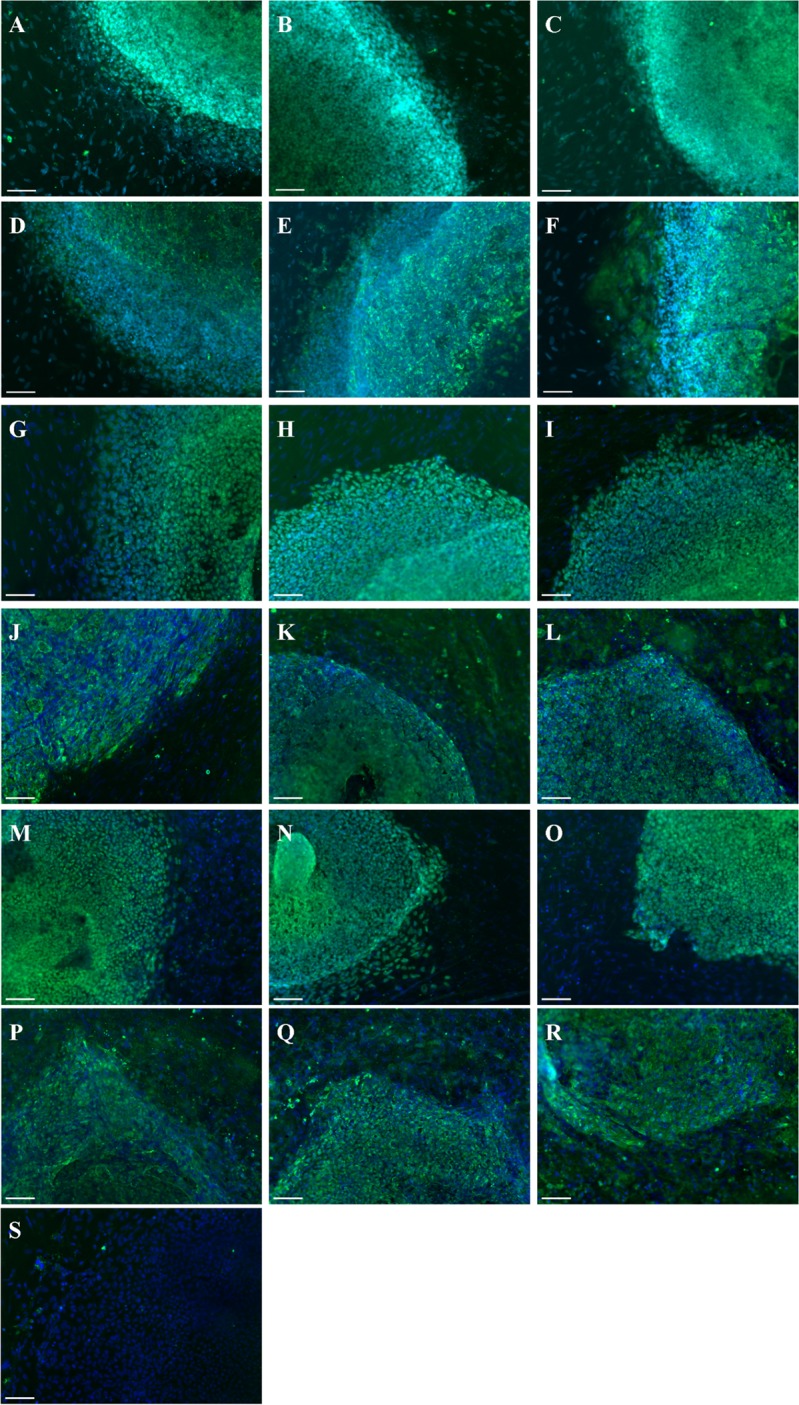
Generation of iPSC lines from FRDA-patients Immunostaining of FA6 CL1 (**A**, **D**), CL2 (**B**, **E**), CL3 (**C**, **F**) for OCT4 (**A-C**) and TRA-1-60 (**D-F**); FA8 CL1 (**G**, **J**), CL2 (**H**, **K**), CL3 (**I**, **L**) for OCT4 (**G-I**) and TRA-1-60 (**J-L**); FA9 CL1 (**M**, **P**), CL2 (**N**, **Q**), CL3 (**O**, **R**) for OCT4 (**M-O**) and TRA-1-60 (**P-R**). (**S**) Negative isotype. Cells were counterstained with DAPI (blue). Scale bars: 50 μm.

**Figure 2:**
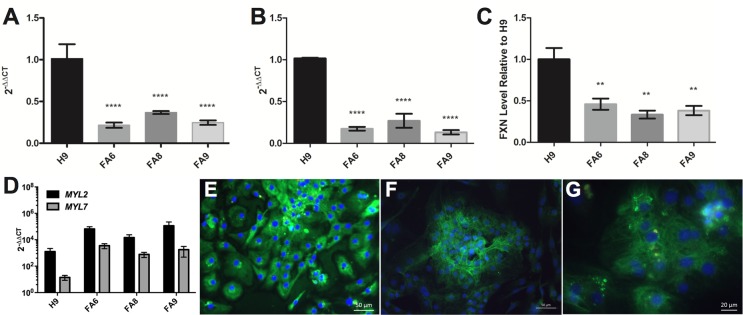
FRDA-iPSCs and - cardiomyocytes retain low levels of FXN and are mainly of ventricular phenotype (**A, B**) qPCR and (**C**) dipstick analysis showing low levels of FXN mRNA (**A, B**) and protein (**C**) in undifferentiated cells (**A**) and their cardiac derivatives (**B**, **C**). Significance was assessed by comparing FRDA-iPSCs to undifferentiated H9 controls (**A**) or FRDA-iPSC derived cardiomyocytes to H9 derived cardiomyocyte controls (**B, C**). One-way ANOVA followed by Bonferroni's multiple comparison test, ** p<0.01, **** p< 0.0001. (**D**) qPCR analysis of cardiomyocytes showing significantly higher expression of *MYL2* than *MYL7* across all cell lines (p<0.05, paired t-test). (**A-D**) Data are mean ± SEM of combined clones or 3 individual experiments, normalized to *ACTB* and relative to undifferentiated cells (**A, B, D**) or normalized to the control line cardiomyocyte (**C**). (**E-G**) Representative images of FA6- cardiomyocytes (**E**), FA8- cardiomyocytes **(F)** and FA9- cardiomyocytes **(G**) for MYL2/MLC2v (green), MYL7/MLC2a (red, weak or absent) and counterstained with DAPI (blue).

We used nucleofection to deliver episomal vectors containing OCT4, SOX2, KLF4, L-MYC, LIN28, shRNA against p53 and eGFP into fibroblasts. Pluripotent clones were expanded and three clones were selected for each patient (CL1-3). All clones expressed the pluripotency markers OCT4 and TRA-1-60 (Fig.1A-S). The intron 1 *FXN* GAA expansions were measured for all fibroblasts and iPSC clones (Table 1, Suppl. Fig. 1). As reported for other FRDA iPSCs [16–18], we observed similar repeat numbers as well as contractions and expansions for all lines, with slight variations between clones of the same line (Table 1). Importantly, the patient-derived iPSC lines each maintained the reduced *FXN* mRNA expression that is characteristic of FRDA, when compared to control cells (Fig. 2A). The iPSCs were karyotypically normal (data not shown), and pluripotent, being able to differentiate into cells of the three germ layers as assessed by embryoid body (EB) formation (Suppl. Fig. 2–4).

**Table 1:** GAA repeats (GAA1/GAA2) GAA1: smaller allele repeats; GAA2: longer allele repeats. F: Female, M: Male. FARS: Friedreich Ataxia Rating Scale.

	FA6 (F, FARS: 96.5)	FA8 (M, FARS: 64.5)	FA9 (M, FARS:118)
**Patient, predicted**	1077/1077	476/545	733/943
**Fibroblasts**	854/247	481/576	788/109
**CL1**	893/281	579	963
**CL2**	887/273	593/323	980/383
**CL3**	980/294	576/315	991

All lines were differentiated into cardiomyocytes using a small-molecule based approach [21]. In basal conditions, FRDA- cardiomyocytes were not hypertrophic, as assessed by the absence of nuclear localisation of NFATC4 (Suppl. Fig. 5), a nuclear translocation known to be a marker of hypertrophy [22, 23]. The FRDA- cardiomyocytes retained low levels of FXN mRNA and protein (Fig. 2B, C). Phenotypes were assessed at day 35. At this stage, transcripts of *MYL2* were significantly higher than *MYL7* across all cell lines (Fig. 2D). The cardiomyocytes expressed MYL2/MLC2v protein and MYL7/MLC2a was rarely observed (Fig. 2E-G). Taken together, these data suggest a ventricular phenotype in the cardiomyocytes that were generated with minimal atrial cardiomyocytes for all lines (Fig. 2D-G) [19, 24].

### FRDA-iPSC-derived cardiomyocytes display phenotypic abnormalities

**Figure 3:**
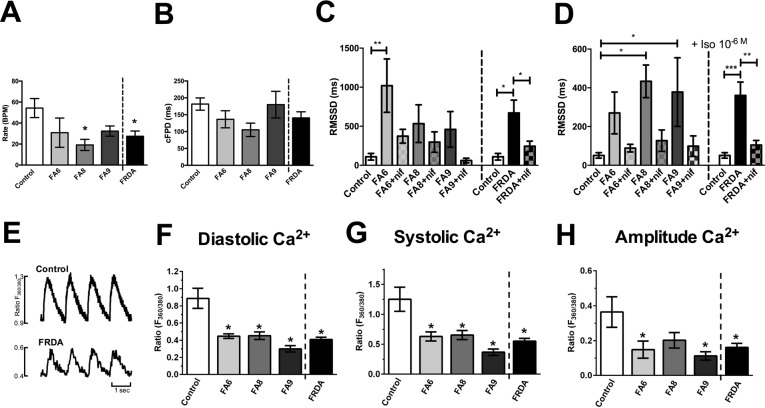
Assessment of phenotypes in FRDA iPSC-derived cardiomyocytes **(A)** Beating rates (beats per minute, BPM), **(B)** corrected extracellular field potential durations (cFPD) and **(C, D)** the root of the mean of the sum of the square of the difference in the RR interval (RMSSD) (ms). Data shows RMSSD at basal (**C**) and 10^−6^M (**D**) isoprenaline ± nifedipine (NIF). **(E)** Representative Ca^2+^ transients. **(F)** Diastolic, **(G)** systolic and **(H)** amplitude Ca^2+^ in Control and FRDA- cardiomyocytes. (**A-D**) Data are mean ± SEM of combined values of 3 clones of each line (n=3 per line) and as a pool of all 3 clones of all FRDA lines (n=9 FRDA independent samples) and control cardiomyocytes (n=6). (**F-H**) Data are mean ± SEM of combined values of control cardiomyocytes (n=5), all 3 clones of each FRDA line (n=8-14 per line) and as a pool of all 3 clones of all FRDA lines (n=35 FRDA independent samples). Statistics: (**A-D, F-H**) One-way ANOVA followed by Bonferroni's multiple comparison test, * p<0.05, **, p<0.01, ***, P<0.001.

The Multi Electrode Array (MEA) revealed electrophysiological anomalies in the different FRDA lines, with consistent effects in all three clones of all FRDA lines. All FRDA lines showed similar basal beat rates (30-40 beat per minute, Fig. 3A) which were slower than control cardiomyocytes. All lines showed similar corrected extracellular field potential durations (cFPD, Fig. 3B). We assessed the root of the mean of the sum of the square of the difference in the RR interval (RMSSD), that measures the variation in a consecutive series of intervals between field potentials (Fig. 3C, D). FRDA-iPSC-derived cardiomyocytes showed significantly increased RMSSD under basal conditions and when treated with isoprenaline (10^−6^ M, Fig. 3C, D). Elevated RMSSD in cultured cardiomyocytes, including those derived from PSCs, is indicative of Ca^2+^ handling abnormalities. When FRDA- cardiomyocytes were examined under basal conditions or following treatment with isoprenaline, application of nifedipine (10^−8^ M), which partially blocks L-type Ca^2+^ channels, prevented an increase in RMSSD, thus confirming that Ca^2+^ is responsible for the abnormal beat rate variability in line with other studies on PSC-derived cardiomyocytes. In contrast, blocking K^+^ channels with TEA (10^−8^ M) did not modify the effect of isoprenaline, with an increase in RMSSD and in cFPD (data not shown). Altogether, these data suggest that FRDA- cardiomyocytes display a significant increase in beat rate variability, demonstrating a potential for cardiac dysfunction, compared to the control cardiomyocytes. These data also suggest that impairment in Ca^2+^ handling is responsible for the observed electrophysiological phenotype. This was confirmed by assessing Ca^2+^ transients. In the FRDA-cardiomyocytes significantly lower diastolic and systolic Ca^2+^ levels and reduced transient amplitude signals were observed compared with control cardiomyocytes (Fig. 3 E-H). Collectively, our data demonstrates a Ca^2+^ handling impairment in the FRDA cardiomyocytes.

## DISCUSSION

Our data reveal electrophysiological anomalies in FRDA iPSC derived - cardiomyocytes, with an increase beat rate variability. As partial inhibition of L-type Ca^2+^ channels with nifedipine abolished this electrophysiological phenotype, it demonstrates that the electrophysiological impairment is due to Ca^2+^ handling abnormalities. As cFPD were not increased in the FRDA iPSC-derived cardiomyocytes, Ca^2+^ overload is unlikely. This was confirmed with measurement of Ca^2+^ cycling, which identified that FRDA cardiomyocytes show low Ca^2+^ transients. Yet, it is difficult to reconcile how the increased beat rate variability is remedied by the Ca^2+^ channel blocker nifedipine, when the Ca^2+^ transients suggest that cytosolic Ca^2+^ levels were already lower than in the controls. It is feasible that in the context of mitochondrial dysfunction in FRDA, ATP-dependent ion channels, particularly sarco/endoplasmic reticulum Ca^2+^-ATPase, may lack the required ATP for proper functioning, leading to reduced Ca^2+^ storage in the sarcoplasmic reticulum and therefore reduced Ca^2+^-induced Ca^2+^ release during an action potential. There have been two previous reports describing phenotypes in FRDA iPSC-derived cardiomyocytes, including Ca^2+^ handling deficiencies, albeit in the presence of exogenous iron [19, 24]. Yet, whilst iron accumulation is generally regarded as a common feature of FRDA pathogenesis, there is scant evidence of iron accumulation in the heart [25]. The data reported here provide the first demonstration of a phenotypic abnormality in cardiomyocytes derived from FRDA-iPSCs without such artificial iron treatment or external stimulus. This suggests that reduced FXN causes dysfunction in cardiomyocytes prior to events such as accumulation of iron. The establishment of a phenotype, itself reversible by selective treatment with nifedipine, now forms a platform to screen molecules known to modify *FXN* and directly assess their impact on human FRDA- cardiomyocytes, and hence contribute to the discovery of specific treatments for FRDA. Indeed, cardiomyocytes derived from patient iPSCs show clear phenotypic abnormalities, consisting of an increase in beat rate variability and reduced Ca^2+^ transients. Although not assessed here, mitochondrial oxidative phosphorylation (OXPHOS) deficiencies, which are reported in FRDA [26], can reduce mitochondrial Ca^2+^ uptake and ATP production [27, 28]. It has also been suggested that mitochondrial OXPHOS defects in FRDA could be an indirect consequence of oxidative stress [29]. Given the impact of reduced Ca^2+^ levels on mitochondrial function, our data suggests that Ca^2+^ handling defects could also contribute to the perceived OXPHOS deficiencies. Treatment of FRDA- cardiomyocytes with nifedipine improved phenotypes detected by MEA. Whilst it may seem contradictory that partially inhibiting L-type Ca^2+^ channels improves cardiomyocytes with low Ca^2+^ levels, it is possible that nifedipine is acting on other Ca^2+^ channels independently [30]. This may either mask or restore the Ca^2+^ levels in the cardiomyocytes [31]. Further in-depth investigations directed at Ca^2+^ handling machinery of FRDA iPSC-derived cardiomyocytes should unravel the mechanisms behind these phenotypes and identify therapeutic targets.

It might be counterintuitive to relate an increased RMSSD in cultured cardiomyocytes to patient phenotypes. Indeed, patients presenting with cardiomyopathy, including FRDA patients, demonstrate reduced heart rate variability compared to healthy individuals [32]. However, these measures in patients relate more to the function of the autonomic nervous system rather than the function of cardiomyocytes. To our knowledge only two studies have assessed Ca^2+^ levels in post-mortem FRDA hearts, both describing elevated right ventricle Ca^2+^ levels, and low-to-normal left ventricle levels in the earlier report versus somewhat elevated left ventricle Ca^2+^ in a recent report [25, 33]. It is however feasible that the FRDA-iPSC derived cardiomyocytes are useful in identifying pathophysiology underlying or preceding the cardiomyopathy observed in FRDA. Importantly, our data clearly indicates that FRDA iPSC- derived cardiomyocytes can be used for screening of compounds able to alter or reverse phenotypes, in human cells, hence providing a novel and unique tool for FRDA research.

## METHODS

### Ethics Committee approvals

All experimental work performed in this study was approved by the Human Research Ethics Committees of the University of Melbourne (0829937, 0605017, 1545383, 1545394) meeting the requirements of the National Health & Medical Research Council of Australia (NHMRC) and conforming to the Declarations of Helsinki.

### Biopsies

The biopsy measured approximately 2–3 mm in diameter and was taken via a needle from the inside of the forearm by a qualified clinician. The risks associated with a skin biopsy are small, however the risks of bleeding and infection were minimised through careful technique, the use of antiseptics and sterile instruments. Minimal pain was experienced at the site once the anaesthetic had worn off and none of the individuals reported any complications following this procedure.

### iPSC Generation

iPSCs were generated using skin fibroblasts obtained from FRDA (FA6, FA8 and FA9) and control subjects over the age of 18 years by an episomal method as described previously [34]. Reprogramming was performed on passage 8-10 fibroblasts by nucleofection with episomal vectors expressing OCT4, SOX2, KLF4, L-MYC, LIN28 and shRNA against p53 [35].

### Maintenance of pluripotent stem cells (PSCs) and cardiomyocyte differentiation

The FRDA-iPSC lines FA6, FA8, FA9 and the control human embryonic stem cell (hESC) line H9 (WiCell) [36] were maintained in the undifferentiated state using TeSR-E8 medium (Stem Cell Tech). H9 was used as a control as the line is very well described; H9-derived cardiomyocytes are structurally and functionally similar to iPSC-derived cardiomyocytes [23, 37, 38], and have been used as a control for overexpression of mutant *MYH7* for modelling cardiomyopathy [23]. hESCs are known to be molecularly and functionally equivalent to iPSCs [39], hence can serve as adequate controls to iPSCs. Embryoid bodies (EB) were obtained as described [40]. In the functional MEA experiments, the control iPSC line, iPSC(Foreskin)-2 [41] was also used, in order to ensure that variations observed between FRDA iPSCs lines and H9 cells were not peculiar to the latter. Differentiation into cardiomyocytes was achieved using a small molecule-based approach, with initial exposure to GSKβ inhibitors (1 µM BIO, 3.5 µM CHIR99021) for 2 days, followed by addition of XAV939 (2 µM) and KY02111 (10 µM) from days 3-8, in IMDM containing 4 mM L-glutamine, 25 mM HEPES, 1% non-essential amino acids, 4 mg/mL human albumin, 100 µM 2-mercaptoethanol, 25 U/mL penicillin, 25 µg/mL streptomycin (all from Thermo Fisher Scientific) and 120 µg/mL L-ascorbic acid sesquimagnesium salt (Sigma Aldrich) [21]. Following two weeks of differentiation in adherent culture, cells were harvested and grown as floating spheres for an additional two weeks in cardiac differentiation medium containing 0.4 mg/mL albumin and 12 µg/mL L-ascorbic acid sesquimagnesium salt. For subsequent work, cells were dissociated with a protease mixture containing 0.1% collagenase I (Wako Pure Chemicals), 0.25% trypsin and 1 U/mL DNase I (Thermo Fisher Scientific) in a buffer consisting of 116 mM NaCl, 20 mM HEPES, 12.5 mM NaH_2_PO_4_, 5.6 mM glucose, 5.4 mM KCl, 0.8 mM MgSO_4_, pH 7.35 (all from Sigma Aldrich). Dissociated cardiomyocytes were plated as monolayers on laminin- or Matrigel-coated plates or slides in albumin- and ascorbic acid-free medium. To assess differentiation, we performed qPCR and/or immunostaining of the cardiac markers actin alpha cardiac muscle 1 (ACTC1), troponin T type 2 (TNNT2), troponin I type 3 (TNNI3) and NK2 transcription factor related locus 5 (NKX2.5) to assess presence of cardiomyocytes, and atrial and ventricular myosin light chain 2 (MLC2a/ MLC2v) to assess the proportion of atrial and ventricular cardiomyocytes. TNN and ACTC staining were also used to assess cardiomyocyte morphology. Nuclear factor of activated T cells 4 (NFATC4) was used to assess the presence of hypertrophy in the cardiomyocytes. A visual assessment of beating cells further confirmed the cardiac phenotype.

### Real-time quantitative RT-PCR

Total RNA was extracted from cells using the RNeasy Mini Kit (Qiagen, Hilden, Germany), converted to cDNA using High Capacity cDNA Reverse Transcriptase Kit (Applied Biosystems, Foster City, CA). Q-PCRs were carried out using TaqMan Universal master mix and the 7900HT Fast Real-Time PCR system using TaqMan gene expression assay for *FXN* (Hs00175940_m1), *MLC2a*/*MYL7* (Hs01085598_g1), *MLC2v*/*MYL2*, (Hs00166405_m1), *ACTC1* (Hs01109515_m1), *TNNT2* (Hs00165960_m1), *TNNI3* (Hs00165957_m1), *NKX2.5* (Hs00231763_m1), Glyceraldehyde 3-phosphate dehydrogenase (Human *GAPDH*, Hs99999905_m1), beta-actin (*ACTB*; Hs99999903_m1) (all from Applied Biosystems). The relative quantitation was achieved by applying the comparative C_T_ method (ΔΔC_T_) whereby the mRNA levels were normalized against the level of *GAPDH* or *ACTB* and the control group was used as the calibrator.

### Immunofluorescence

Cells were fixed with 4% paraformaldehyde (PFA) or ethanol (OCT-4), blocked in 10% fetal calf serum-PBT, and immunostained using the following antibodies: mouse anti-OCT3/4 (Santa Cruz Biotechnology), mouse anti-TRA-1-60 (Millipore), mouse anti-NESTIN (Millipore), rabbit anti-alpha-fetoprotein (AFP, Dako), mouse anti-smooth muscle actin (SMA, R&D systems), mouse anti-ACTC1 (Abcam), rabbit anti-NFATc4 (Santa Cruz), rabbit anti-MYL2 (Proteintech), mouse anti-MYL7 (abcam). Cells were then immunostained with the appropriate conjugated secondary antibodies (Alexa Fluor 568 or 488, Molecular probes-Invitrogen). Nuclei were counter-stained with Hoechst-33342 (Sigma-Aldrich) or DAPI (Invitrogen). Specificity of the staining was verified by the absence of staining in negative controls consisting of the appropriate negative control immunoglobulin fraction (Dako).

### GAA expansion analysis

Genomic DNA for GAA expansion analyses was extracted from fibroblasts and iPSCs using the QIAamp DNA Mini Kit (Qiagen) according to the manufacturer's instructions. The concentration and purity of the genomic DNA were assessed using a Nanodrop1000 spectrophotometer (Thermo Fisher Scientific). The size of the GAA expansion in intron 1 of the *FXN* gene was determined by PCR using the Expand Long Range dNTPack (Roche, Australia) as recommended with 20 ng template DNA, 0.4 μM of EXP-Bam-F 5′AAGGAAGTGGTAGAGGGTGTTTCACGAGGA3′ and EXP-Bam-R 5′TTTGGATCCAACTCTGCTGACAACCCATGCTGTCCACA3′ primers and 1x Q solution (QIAGEN, Australia). PCR products were electrophoresed on a 1% (w/v) agarose, 1x TAE gel alongside standard DNA markers (200 bp ladder, Promega). Size determination was performed using GeneTools software from SynGene, Synoptics (In Vitro Technologies). The positive control (BAC clone RP11-265B8) and non-expanded alleles in the normal range yielded an 810 bp fragment. The hESC H9, BG01V (ATCC) and the human fibroblast feeders WS1 (ATCC) were included as negative controls for *FXN* expansion.

### Dipstick of FXN expression

Dipstick assay for FXN (Mitosciences) was performed as per the manufacturer's instructions [42]. This method allows the quantification of FXN by using two specific monoclonal antibodies against different antigens of FXN, in a sandwich ELISA assay. Briefly, one capture antibody bound to a nitrocellulose membrane captures FXN, and a detector antibody conjugated to gold provides a signal when bound to the complex of capture antibody-FXN-detector antibody. Levels of FXN are then detected and quantified by an increased signal intensity.

### Electrophysiological characterization

Electrophysiological measurements of cardiomyocytes seeded onto laminin-coated 60 electrodes MEA plates were performed by MEA (Multichannel Systems, Reutlingen, Germany) as previously described [40]. Cells were incubated at 37°C in cardiac differentiation medium without albumin and ascorbic acid. Basic receptor and ion channel function were assessed by treatment of cardiomyocytes with nifedipine (inhibitor of L-type Ca^2+^ channel) and isoprenaline (β-adrenergic receptor agonist) (Sigma-Aldrich). Extracellular field potentials were recorded at baseline and 1 minute after addition of drugs. Data were analyzed offline with MC Rack version 4.3.5 software for beating rate, inter-beat (RR) interval and extracellular field potential duration (FPD). RR interval was defined as the time elapsing between two consecutive beats, and FPD was defined as the time interval between the initial deflection of the field potential and the maximal local T wave. To avoid the influence of beat frequency on FPD, FPD measurements were normalized (corrected FPD, cFPD) with the Bazett's correction formula: cFPD = FPD/√(RR interval) [43]. Data were expressed as percentage change from baseline with baseline set to 100%. Root of the mean of the sum of the square of the difference in the RR interval (RMSSD) was also assessed.

### Intracellular Ca^2+^ and contractility measurements

Intracellular Ca^2+^ was measured by microfluorimetry (Ionoptix MA, USA). Cells were loaded with the Ca^2+^ fluorescent dye FURA-2/AM (5 μM, 10 min incubation at room temperature, Molecular Probes). They were placed in a chamber mounted on the stage of a Motec AE31 inverted fluorescence microscope immersed in medium (IMDM without phenol red, containing 4 mM L-glutamine, 25 mM HEPES, 1% non-essential amino acids, 25 U/mL penicillin and 25 µg/mL streptomycin; the total concentration of Ca^2+^ in the medium was 1.5 mM), at room temperature and stimulated to contract at 1Hz. Excitation light at 340 nm and 380 nm was provided by a 75-watt xenon lamp and filter wheel. Emitted fluorescence (510 nm) was recorded by a photomultiplier tube, with the output current converted to voltage and digitized for subsequent analysis. Background correction was undertaken at the completion of each cell recording and incorporated into analysis protocol. The following Ca^2+^ parameters were measured: diastolic Ca^2+^, systolic Ca^2+^, amplitude of the Ca^2+^ transient and time constant of decay of the Ca^2+^ transient (tau).

### Statistical analysis

Data are expressed as mean ± standard error of the mean (SEM). All statistical analyses and graphical data were generated using Graphpad Prism software (v5.04, www.graphpad.com). Statistical methods utilized were one-way ANOVA followed Tukey's or Bonferroni's multiple comparisons test and t-test. Statistical significance was established as *p<0.05, **p<0.01, ***p<0.001, ****p<0.0001.

## SUPPLEMENTARY MATERIAL


