# Supplementary material for: Friedreich's ataxia induced pluripotent stem cell-derived cardiomyocytes display electrophysiological abnormalities and calcium handling deficiency
Source: Aging (Albany NY). 2017 May 30;9(5):1440–9. doi: 10.18632/aging.101247 (PMC5472743; doi:10.18632/aging.101247)
Supplement: Supplementary file 1 [file aging-09-1440-s001.pdf]

SUPPLEMENTARY MATERIAL

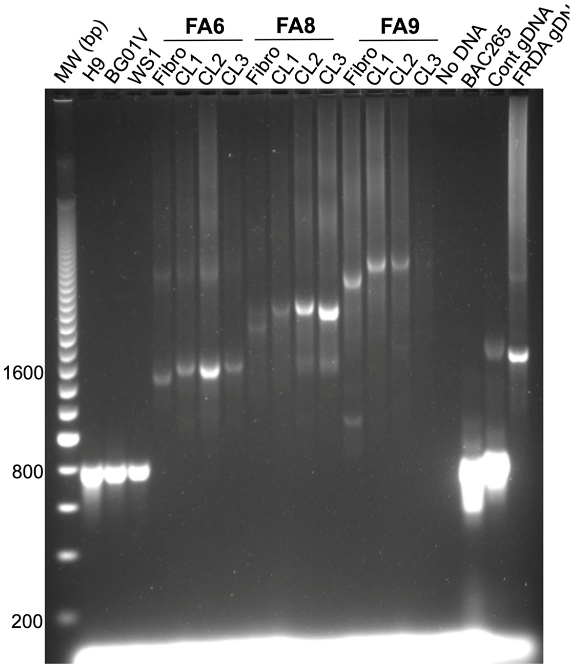

Supplementary Figure 1. GAA expansion PCR. The size of the GAA expansion was determined in control (H9, BG01V, WS1) and FRDA cells (FA6, FA8 and FA9) by comparison to standard DNA markers (MW). Genomic DNA isolated from fibroblasts (Fibro) and iPSC clones (CL) 1-3 were used in long range PCR of the first intron of FXN. Non-expanded alleles yield a product of 810 bp. Positive (Human embryonic stem cell lines H9 and BG01V, human fibroblast WS1, BACRP11-265B8 DNA containing the FXN gene, Control genomic (g)DNA and FRDA gDNA) and negative controls (no DNA) were included.

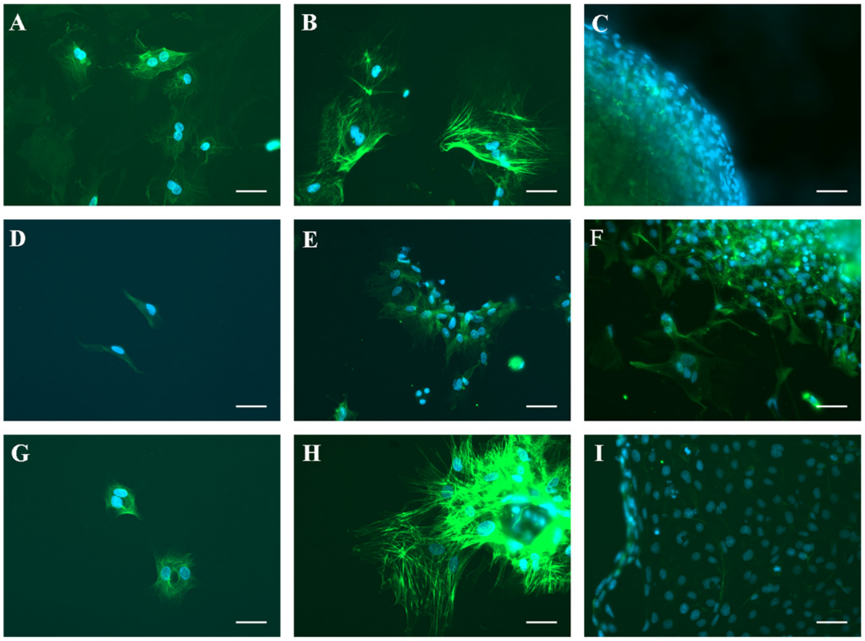

Supplementary Figure 2. Representative germ layer immunostaining of FA6-EBs. FA6 Clone 1-3 (**A**-**C**, **D**-**F**, **G**-**H**) demonstrate pluripotency by positive staining for markers of each embryonic germ layer; endoderm (AFP; **A**, **D**, **G**), mesoderm (SMA; **B**, **E**, **H**) and ectoderm (nestin; **C**, **F**, **I**). Scale bars = 50 µm.

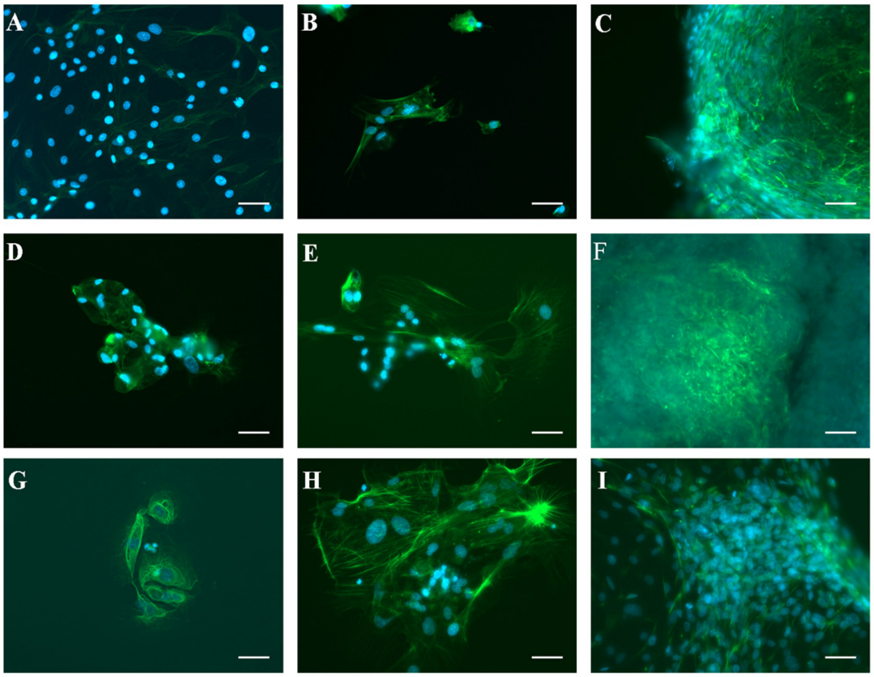

Supplementary Figure 3. Representative germ layer immunostaining of FA8-EBs. FA8 Clone 1-3 (**A**-**C**, **D**-**F**, **G**-**H**) demonstrate pluripotency by positive staining for markers of each embryonic germ layer; endoderm (AFP; **A**, **D**, **G**), mesoderm (SMA; **B**, **E**, **H**) and ectoderm (nestin; **C**, **F**, **I**). Scale bars = 50 µm.

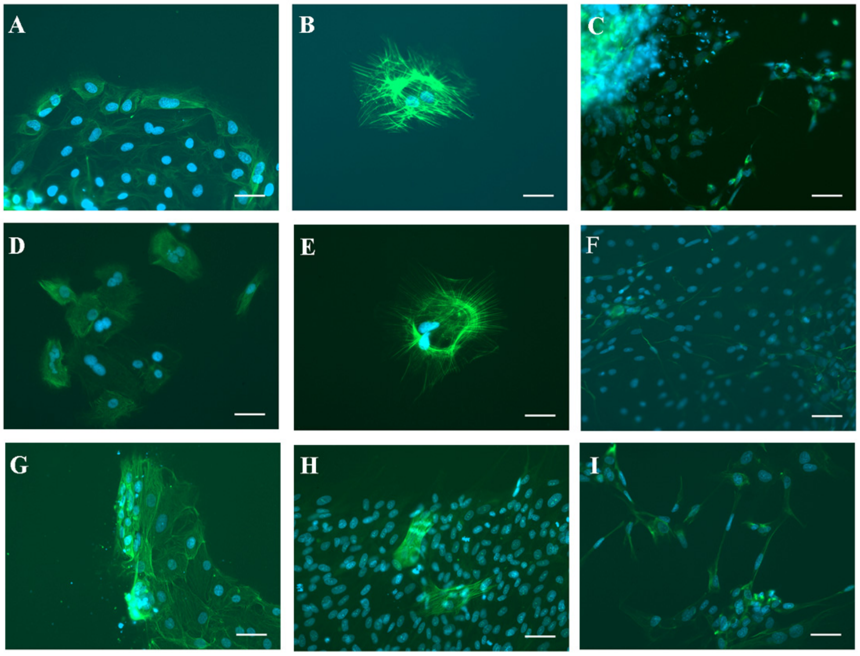

Supplementary Figure 4. Representative germ layer immunostaining of FA9-EBs. FA9 Clone 1-3 (**A**-**C**, **D**-**F**, **G**-**H**) demonstrate pluripotency by positive staining for markers of each embryonic germ layer; endoderm (AFP; **A**, **D**, **G**), mesoderm (SMA; **B**, **E**, **H**) and ectoderm (nestin; **C**, **F**, **I**). Scale bars = 50 µm.

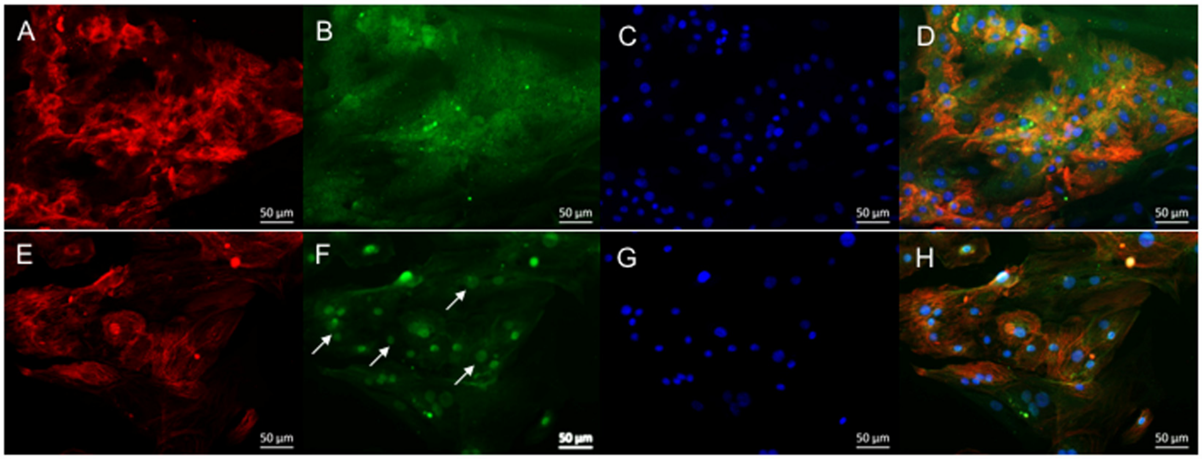

Supplementary Figure 5. Assessment of hypertrophy in FRDA-cardiomyocytes. FA8-cardiomyocytes 35 days post-differentiation untreated (**A**-**D**) or treated with 1µM isoprenaline to induce hypertrophy (**E**-**H**), immunostained for troponin (**A**, **E**); NFATc4 (**B**, **F**), counterstained with DAPI (**C**, **G**) and merged (**D**, **H**). Nuclear localisation of NFATc4 is indicative of hypertrophy and some are illustrated with arrows. Data presented on FA8 cells are representative of all cell lines tested.
